# S1 Guideline of the German Society for Neurogastroenterology and Motility (DGNM) on Functional Dyspepsia (FD), a Disorder of Gut–Brain Interaction (DGBI) [English Language Edition]

**DOI:** 10.1155/grp/2610765

**Published:** 2026-05-05

**Authors:** M. Storr, V. Andresen, T. Frieling, J. M. Gschossmann, J. Keller, J. Langhorst, C. Pehl, A. Stengel, J. Tebbe, K. Wiemer, A. Madisch, M. Stengel

**Affiliations:** ^1^ Medical Clinic II of the Ludwig Maximilian University, Munich, Germany; ^2^ Center for Endoscopy, Internal Medicine Center Gauting-Starnberg, Starnberg, Germany; ^3^ Private Practice Gastroenterology, Medizinicum, Hamburg, Germany; ^4^ Helios Klinikum Krefeld, Internal Medicine With Gastroenterology, Hepatology, Infectious Diseases, Neurogastroenterology, Gastrointestinal Oncology, Hemato-Oncology and Palliative Medicine, Krefeld, Germany; ^5^ Department of Internal Medicine, Klinikum Forchheim-Fränkische Schweiz, Forchheim, Germany; ^6^ Medical Clinic, Israelitisches Krankenhaus, Hamburg, Germany; ^7^ Clinic for Integrative Medicine and Naturopathy at the Klinikum am Bruderwald, Sozialstiftung Bamberg, Bamberg, Germany; ^8^ Endowment Chair for Integrative Medicine, University of Duisburg-Essen, Klinikum Bamberg, Bamberg, Germany, uni-due.de; ^9^ Medical Clinic, Krankenhaus Vilsbiburg, Vilsbiburg, Germany; ^10^ Clinic for Psychosomatic Medicine and Psychotherapy, Klinikum Stuttgart, Stuttgart, Germany, klinikum-stuttgart.de; ^11^ Department of Internal Medicine VI, Clinic for Psychosomatic Medicine and Psychotherapy, University Hospital Tübingen, Tübingen, Germany, uni-tuebingen.de; ^12^ German Center for Mental Health (DZPG), Tübingen, Germany; ^13^ Department of Gastroenterology & Infectiology, Klinikum Lippe, Medical School and University Medical Center OWL, Bielefeld University, Bielefeld, Germany, uni-bielefeld.de; ^14^ Medical Clinic II-Clinic for Gastroenterology, Knappschaft Kliniken Kamen, Kamen, Germany; ^15^ Center for Gastroenterology Bethanien, Agaplesion Krankenhaus Bethanien, Frankfurt, Germany; ^16^ Medical Clinic, SRH Klinik Sigmaringen, Sigmaringen, Germany

## Abstract

Functional dyspepsia (FD) is common and classified as a disorder of gut–brain interaction (DGBI). The prevalence is estimated around 10% of the population. Diagnosis is based on symptoms, which are classified according to the Rome IV criteria, in combination with diagnostic procedures that may include laboratory testing, *Helicobacter pylori* testing, upper gastrointestinal endoscopy, abdominal ultrasound, and other examinations, depending on the severity, duration, and presence of alarm symptoms. Therapeutic procedures include psychoeducation, dietary counseling, mind–body procedures, psychotherapy, and medication. The S1 guideline summarizes the current state of knowledge and allows a targeted approach based on the currently available medical evidence.

## 1. Epidemiology and Risk Factors

The term “functional dyspepsia” (FD) refers to recurring or chronic symptoms of the upper abdomen that cannot be attributed to an organic disease by using standard diagnostic procedures [1]. In addition to irritable bowel syndrome (IBS), FD represents a central syndrome within functional gastrointestinal diseases (FGID) or disorders of gut–brain interaction (DGBI), respectively. The high prevalence of FGID was recently confirmed in a large multinational study. Of the more than 70,000 people included in the survey, 40% showed clear evidence of FGID, according to the Rome IV criteria [2]. Epidemiological studies suggest that roughly 10% of the population meet the criteria for FD [3].

Various risk factors for the occurrence of FD have been identified to date: Incidence of FD is increased in the female population [2, 3]. Previous gastrointestinal infections predispose to functional symptoms. In a meta‐analysis of 19 studies, Futagami et al. observed the development of FD in 9.6% of cases, six or more months after an acute gastroenteritis [4]. Thus, the term postinfectious FD has been established. However, the use of nonsteroidal anti‐inflammatory drugs also represents a risk factor for the development of FD. Shaib and El‐Serag were able to demonstrate this in a multiethnic study population consisting of 203 employees of the Houston Veterans Affairs Medical Center [5]. Depression represents another comorbidity associated with an increased likelihood of FD. In a trinational study conducted *via* internet survey in the United States, Canada, and Great Britain, Aziz and colleagues were able to establish a negative association between the use of antidepressants and the presence of postprandial distress syndrome (PDS) [6]. In a large study based on data from the Swedish Population Register, Aro and colleagues documented a 7.6‐fold increase in the risk of developing FD within 10 years when an anxiety disorder was initially present. Interestingly, in this study, the diagnosis of depression had no significant effect on the subsequent development of FD [7].

1.1 Statements–Recommendations Functional Dyspepsia, the main representative of functional gastrointestinal disorders affecting the upper gastrointestinal tract, is highly prevalent.

1.2. Various risk factors have been identified for the development of FD, but their influence on the pathophysiological processes leading to the clinical presentation of FD is only partially understood to date.

## 2. Pathophysiology

### 2.1. Disorders of Gastroduodenal Motility

Disturbed gastric accommodation and/or disturbed gastric emptying, which in turn can be accelerated or delayed, is found in up to 70% of FD patients [8]. Approximately 30% of patients exhibit delayed gastric emptying of solid food [9], which has traditionally been considered an important pathomechanism in patients with PDS [10]. However, the relationship between gastric emptying velocity and symptoms is weak, and the prevalence of delayed gastric emptying in PDS and epigastric pain syndrome (EPS) does not differ significantly. Differentiating FD from gastroparesis can also be difficult. The recent European guideline attempts to do this through symptomatology, whereby patients with gastroparesis, by definition, have (significantly) delayed gastric emptying and primarily suffer from nausea and vomiting [11, 12]. However, recent studies show relevant shifts between both clinical pictures over time [13]. Impaired gastric accommodation in response to a meal may be a key mechanism for the development of symptoms in FD, especially in PDS, where up to 40% of affected individuals exhibit this disorder [14]. In contrast, in more recent studies, dysfunction of gastric accommodation was found in approximately equal frequencies for PDS, EPS, and mixed disease patterns, so that there does not seem to be a clear connection between the type of disorder and the symptoms [10]. However, it has been shown that patients with pronounced and combined motility disorders had a particularly severe symptomatology [8].

### 2.2. Disorders of Gastroduodenal Sensitivity

Increased visceral sensitivity generally plays an important pathophysiological role in FGID [15]. In FD, increased visceral sensitivity can cause normal digestive processes such as gastric filling after a meal being perceived as unpleasant or painful. Hypersensitivity to gastric distension is observed in 37% of FD patients [16]. In addition, hypersensitivity to chemical stimuli such as gastric acid and duodenal fat is observed. Gastroduodenal hypersensitivity appears to correlate with the severity of symptoms [15], but no clear differences between PDS and EPS have been found so far [10].

### 2.3. Impaired Intestinal Barrier Function and Immunological Changes

Recent studies show that impaired duodenal barrier function and mild inflammatory changes in the duodenal mucosa contribute to the pathophysiology of FD [17]. As a sign of gastroduodenal immune activation, patients with FD have increased numbers of eosinophilic granulocytes and, coupled with this, increased numbers of mast cells [18–20]. The first antibodies against these are in clinical trials [21], and the efficacy of proton pump inhibitors (PPIs) in FD could also depend, at least in part, on a reduction of mucosal eosinophilia [20].

In patients with postinfectious symptoms, changes in mucosal lymphocyte subpopulations and mucosal barrier dysfunction are found; the latter is most likely caused by immunological phenomena but may also contribute to immunological disturbances [22].

In patients with IBS, confocal laser endomicroscopy has shown that atypical food allergies can cause acute damage within the intestinal barrier with fluid leakage [23]. A higher density of “epithelial gaps” compared to healthy subjects has already been demonstrated using this method in patients with FD [24]. Whether there is a direct correlation with nutrient contact remains unresolved.

### 2.4. Altered Microbiota/Gastrointestinal Infections

A total of 10%–15% of patients with chronic dyspeptic complaints have chronic *Helicobacter pylori* (*HP*) infection without ulcers or high‐grade gastritis. In roughly 10% of these, symptoms disappear after eradication therapy. However, this subgroup is then classified as *HP*‐associated dyspepsia and no longer as FD [25]. In general, gastrointestinal infections, including COVID‐19 [26], can also trigger FD. According to a meta‐analysis, the prevalence of FD following gastroenteritis is approximately 10%, and the risk is increased by a factor of 2.5 compared to controls [4]. Studies also show that, regardless of the type of infection, the density of bacterial colonization of duodenal mucosal samples in FD correlates directly with the extent of postprandial symptoms and indirectly with quality of life [22]. Other research groups found reduced diversity within the small intestinal microbiota, which correlated with increased intestinal permeability [27].

### 2.5. Genetic Predisposition

In patients with FD, the GN*β*3‐TT genotype was observed more frequently compared to controls, and the cholecystokinin (CCK)‐A receptor CC genotype was observed significantly less frequent [28], but the data in this context are inconsistent [29]. Affected individuals could therefore not only be predisposed to dysfunctional hormone regulation of gastrointestinal functions but also exhibit alterations in central serotonin metabolism associated with mental disorders.

### 2.6. Biopsychosocial Influencing Factors

Stress can affect gastroduodenal motility, for example, delay gastric emptying and thereby promote dyspeptic symptoms [30]. In addition, acute psychological stress has been shown to increase intestinal permeability, thereby damaging the mucosal barrier and triggering the pathomechanisms described above [31]. However, patients with FD also report heightened psychological symptoms/illnesses such as anxiety, depression, somatization, and neuroticism. These increase when the severity of abdominal symptoms intensifies [32]. Patients with FD are more likely to have experienced abuse or other stressful events. These factors promote pathological central processing of visceral stimuli with heightened vigilance toward (unpleasant) sensations arising in the gastrointestinal tract. Overall, mental disorders and abdominal symptoms form a vicious circle, which negatively influence each other. However, prospective epidemiological studies show that in at least half of the cases with FGID, abdominal symptoms precede psychological symptoms and could possibly also be the cause of the psychological disorders [22].

### 2.7. Pathophysiological Model

Further studies are needed to clarify how these factors specifically interact and where new therapeutic approaches may emerge. One hypothesis is that mucosal contact with certain allergens (e.g., microbial antigens or food proteins after acute gastroenteritis) causes mucosal barrier dysfunction and immune activation with mild inflammation in genetically predisposed individuals. As a result, structural and functional changes may occur, particularly in the enteric nervous system, which cause hypersensitivity and motor disorders [33]. Trauma and psychological disorders promote a “pathological” central processing of visceral stimuli and may contribute to the development of FD in a variable extent. The connection of biological, psychological, and social factors can be found in the biopsychosocial explanatory model.

Statements–Recommendations

2.1 The pathophysiology of FD is complex and individual and can be best explained using the biopsychosocial model.

2.2 Relevant pathomechanisms are disordered gastroduodenal motility, visceral hypersensitivity, changes in the mucosal barrier, the immune system and the enteric nervous system, and altered central processing. These disorders are in turn influenced by traumatic life events, stress, concomitant psychological, and genetic factors.

2.3 Gastrointestinal infections can trigger FD.

2.4 Clear differences in the pathophysiology of PDS and EPS have not yet been demonstrated.

## 3. Diagnosis

### 3.1. Background

The diagnosis of FD is based on two conditions: firstly, the typical symptoms and secondly, exclusion of other organic diseases of the upper gastrointestinal tract that may cause comparable symptoms [28, 34–36].

The definition of FD is symptom based and does not indicate a universally applicable pathophysiology. This also applies to the subgroups, PDS and EPS, which overlap in about 30% of patients [34]. Following consequent diagnostic workup and confirmation of the diagnosis, repeat examinations should be avoided. Re‐evaluation and additional diagnostic procedures may only be necessary in cases of significant changes in symptoms or in treatment‐resistant patients.

### 3.2. Typical Clinical Presentation

Since the symptoms are not specific, the Rome consensus conference was trying to narrow down the diagnostic criteria for FD [34, 37, 38]. However, the symptoms of postprandial fullness, early satiation, epigastric pain, and epigastric burning cannot be clearly separated from symptoms caused by other organic disease and clearly do not correlate with gastric functions. Only the symptom of early satiation showed a correlation with impaired fundus relaxation [39], while gastric emptying time had virtually no correlation with the symptoms. In addition, a recent study indicates that subtype specific therapy was without significant advantage [40]. Other recent studies indicate a paradigm shift and focus the pathophysiology to the duodenal mucosa [41].

Therefore, it is difficult to differentiate between FD and gastroparesis based on symptoms only. Gastroparesis overlaps with FD, especially the PDS subtype [34]. Since delayed gastric emptying can occur in both conditions, it is not always possible to establish a definitive diagnosis in routine clinical practice. In a large prospective registry study, the diagnosis of gastroparesis needed to be changed from gastroparesis to FD in 42% of cases, and from FD back to gastroparesis in 37% of cases, within 1 year [13]. A European position paper therefore proposed focusing on nausea (the most common symptom, occurring in > 95% of cases) and vomiting with subsequent improvement in symptoms as cardinal symptoms of gastroparesis, which generally occur in the context of food intake. In contrast, postprandial fullness, early satiation, epigastric pain, and burning are more likely to indicate FD. This clinical definition of the dominant symptoms (“cardinal symptoms”) may help in differentiating between the two clinical entities and improve the precision of future treatment studies [42, 43].

### 3.3. Exclusion of Other Organic Diseases

#### 3.3.1. Laboratory Tests

The importance of labwork in dyspepsia is unclear [1]. However, not all warning signs can be detected without laboratory analysis.

Preliminary laboratory tests such as blood count, electrolytes, liver and kidney function, erythrocyte sedimentation rate or CRP, and, if necessary, peripheral thyroid parameters can be determined. However, there is no scientific evidence to support their significant role in diagnosing FD.

#### 3.3.2. Ultrasound

According to a systematic review and meta‐analysis, there is only a significant association with colic pain and gallstones detected by ultrasound but not with dyspeptic symptoms [44, 45]. In patients over 60 years of age, presenting with the combination of epigastric pain and weight loss as well as epigastric pain radiating into the back, CT/MR/EUS diagnostics should be considered rather than sonography to exclude pancreatic carcinoma [46, 47].

#### 3.3.3. Probatory Treatment

A meta‐analysis (five RCTs, 1752 patients with dyspepsia) found no significant difference in dyspepsia symptoms between prompt endoscopy and empirical acid suppression with PPIs or histamine‐2 receptor antagonists [47]. However, patient satisfaction was higher among patients who underwent upper endoscopy [48].

#### 3.3.4. Esophagogastroduodenoscopy (EGD)

According to the Rome IV definition, EGD with *HP* testing is mandatory for the diagnosis of FD in patients older than 45–60 years or younger patients with alarm symptoms (weight loss, recurrent vomiting, short medical history in older patients, fever, family history of esophageal/gastric cancer, bleeding, dysphagia, odynophagia, and abdominal mass) [34]. However, the probability of finding an explanation for the dyspeptic symptoms is very low, with 8% ulcers, 20% esophagitis, and less than 1% malignancies. According to meta‐analyses, 70% of patients are still classified as having FD after EGD. In younger patients without alarming symptoms, EGD is not necessary, and probatory treatment with PPIs, prokinetics, or *HP* eradication (“test and treat”) can be carried out.

During diagnostic workup, patients frequently receive the diagnosis of “gastritis” based on the endoscopic and histological findings, even though FD is actually present. The term “gastritis” as a clinical diagnosis should therefore be avoided in favor of the diagnosis of FD, especially since the endoscopic and histological findings of gastritis do not correlate with the patients′ symptoms [49].

A meta‐analysis of seven population‐based endoscopic studies on the recovery of upper GI endoscopy in patients with dyspepsia showed that the most common findings were erosive esophagitis (14%) and gastric (2.5%) or duodenal ulcers (4.9%) [50]. The diagnostic yield of upper endoscopy increases with age. Malignancies are rare in patients under 45 years of age or in patients 50 years of age without warning signs [51, 52]. More than 70% of endoscopically examined patients with dyspeptic symptoms qualify for the diagnosis of FD [1]. Important: FD is also present in the case of histological evidence of type C gastritis, regardless of its severity [34].

#### 3.3.5. *HP* Eradication

All patients with evidence of *HP* infection should be eradicated, since *HP* infection is a risk factor for the development of gastric cancer [53]. However, the prevalence of *HP*‐associated dyspepsia is low and found in only about 10% of *HP*‐positive patients with dyspepsia. A meta‐analysis found a number needed to treat (NNT) of 12.5 to successfully treat a patient with dyspepsia [1].

A meta‐analysis of six studies (2399 patients with dyspepsia) comparing *HP* testing or treatment with prompt endoscopy showed no difference in global dyspepsia symptoms [47]. After 1 year, 74% versus 77% of patients still had symptoms. Patient satisfaction is higher with early endoscopy compared to “test and treat” [48].

#### 3.3.6. Determination of Gastric Emptying Time

The prevalence of delayed gastric emptying in FD is around 30%. However, gastric emptying time does not correlate with symptoms or treatment response. Therefore, determining gastric emptying time without clinical suspicion of gastroparesis is not recommended [1].

#### 3.3.7. 24‐h pH–Impedance Study of the Esophagus

Pathological gastroesophageal reflux is detected in 20%–30% of patients with dyspepsia without heartburn and in up to 50% of patients with epigastric burning. Although only a small group of patients with FD responds to acid‐suppressive therapy, studies do not conclusively demonstrate that esophageal pH measurement or esophageal impedance pH measurement in the FD patient population can identify this subgroup [1].

#### 3.3.8. Small Intestine Diagnostics

Recent studies show that patients with FD also have changes in the duodenal wall, with increased mast cells or eosinophilic granulocytes. The mucosal barrier (permeability disorder; so‐called “leaky gut”) also appears to be impaired. Albeit, in this context, a paradigm shift in the pathophysiology of FD has been postulated [54]. However, these findings have not yet been recommended as diagnostic markers for FD.

#### 3.3.9. Endoscopic Laser Endomicroscopy

In clinical studies, endoscopic confocal laser endomicroscopy is suitable, with limitations, for detecting evidence of small intestine integrity (increased permeability/leaky gut) and immunological food reactions of the duodenal mucosa. This has been shown for IBS and FD [23, 55, 56]. However, there is currently no standardized procedure for endoscopic laser endomicroscopy. Therefore, this test cannot be recommended for clinical diagnostics yet.

#### 3.3.10. Nutrient Drinking Test

Studies have demonstrated reduced volume tolerance in some patients with FD. This can be investigated by the rapid drinking test for liquid food or water, which correlates with clinical symptoms and symptom severity. However, there is currently no standardized test protocol, and this test cannot be recommended for clinical routine.

### 3.4. Differential Diagnoses

The relevant differential diagnoses, the main symptoms and the accompanying symptoms, and the Rome IV diagnostic criteria are listed in Tables 1, 2 and 3.

**Table 1:** Relevant differential diagnoses of FD [57–59].

Condition	Comment
Gastroparesis	Overlap up to 50%, main symptom vomiting
Irritable bowel syndrome	Overlap up to 60%, abdominal pain associated with bowel movements
Gastroesophageal ulcer disease	Found in about 8%
Gastroesophageal reflux disease	FD can be associated with heartburn in up to 50% of cases
Drug side effects (e.g., nonsteroidal anti‐inflammatory drugs)	Detailed medication history, discontinuation attempt
Gastric carcinoma	Less than 1%

**Table 2:** Main symptoms and accompanying symptoms of FD [34, 60].

Main symptoms	Associated symptoms
Epigastric pain	Belching
Epigastric burning	Nausea
Postprandial fullness	Bloating
Early satiation	Heartburn (not being the main symptom)

**Table 3:** Rome IV diagnostic criteria for functional dyspepsia (modified as per [34]).

**One or more of the following:** **► Bothersome** ∗ **epigastric pain.** **► Bothersome** ∗ **epigastric burning.** **► Bothersome** ∗ **postprandial fullness.** **► Bothersome** ∗ **early satiation.** **► Symptom onset at least 6 months before diagnosis.** **► The symptoms should be present in the last 3** months. **► No evidence of structural disease (including gastroscopy) that could explain the symptoms.**	
Epigastric pain syndrome (EPS) Symptoms occur at least 1 day per week plus one or both of the following symptoms: 1. Bothersome∗ epigastric pain. 2. Bothersome∗ epigastric burning. Supporting criteria: 1. Pain may be induced or relieved by eating or may occur during fasting. 2. Postprandial epigastric bloating, belching, and nausea may also be present. 3. The pain does not meet the criteria for biliary pain.	Postprandial distress syndrome (PDS) Symptoms occur at least 3 days per week plus one or both of the following symptoms: 1. Bothersome∗ postprandial fullness. 2. Bothersome∗ early satiation (i.e., strong enough that a normal‐sized meal cannot be eaten). Supporting criteria: 1. Postprandial epigastric pain or burning, epigastric distension, excessive belching, and nausea may be present.
Persistent vomiting likely indicates another disorder. Heartburn is not a dyspeptic symptom but can often coexist. Bowel habit changes are not part of the dyspepsia syndrome. Other digestive symptoms (such as gastroesophageal reflux disease and IBS) may coexist with FD.	

∗Bothersome: symptoms severe enough to interfere with daily activities.

Statements–Recommendations

3.1. The diagnosis of FD is based on the typical symptoms and the exclusion of other organic disease of the upper gastrointestinal tract that may cause comparable symptoms. It follows a clear algorithm.

3.2. In addition to medical history, basic laboratory tests, EGD, and *HP* testing are the diagnostic tests of choice.

3.3. Determination of gastric emptying time and 24‐h pH impedance measurement in the esophagus are not routine diagnostic tests in FD but can be helpful in excluding gastroparesis or gastroesophageal reflux disease.

3.4. In cases of severe weight loss and very restrictive eating behavior, an eating disorder should be ruled out.

## 4. Diagnostic Algorithm

**Figure 1:**
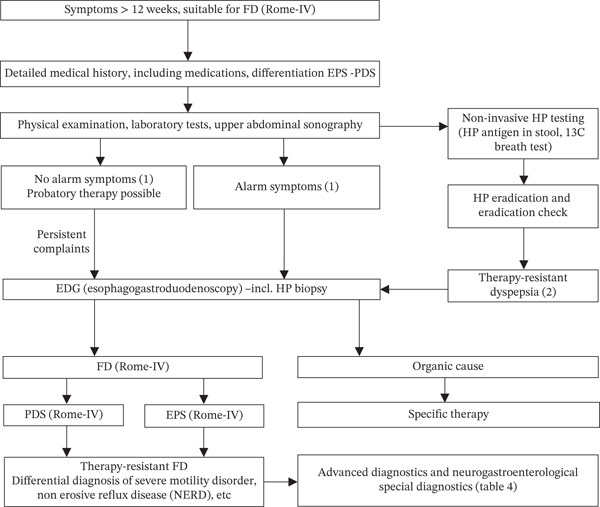
Algorithm for diagnosing functional dyspepsia.

The algorithm for diagnosing FD is shown in Figure 1.

**Explanations for** Figure 1

(1) Alarm symptoms

Age >55 ± 5 years; positive family history of gastric cancer; weight loss; anemia, thrombocytosis, vomiting, dysphagia, upper gastrointestinal bleeding, fever, and palpable abdominal mass. The positive predictive value (PPV) of alarm symptoms is low (0%–11%), on average 1.1% (excluding studies with 0%) [61]. However, the negative predictive value is > 99%. Regarding individual alarm symptoms, the PPV is higher for weight loss than for dysphagia (sensitivity and specificity for esophageal carcinomas or adenocarcinomas of the esophagogastric junction, gastric carcinomas) and lowest for the presence of anemia.

(2) Therapy‐resistant FD

Even though improvement in symptoms may be delayed for up to 6–12 months [1, 47], endoscopy should be performed early (lack of improvement after 4 weeks) after unsuccessful *HP* eradication [47]. The neurogastroenterological differential diagnosis in treatment‐resistant FD is listed in Table 4.

**Table 4:** Extended neurogastroenterological differential diagnosis in treatment‐resistant FD.

Predominant symptoms	Further investigation
Associated reflux symptoms	24‐h pH monitoring, 24‐h pH impedance measurement, and possibly esophageal manometry to rule out nonerosive reflux disease
Especially for upper abdominal bloating	H_2_ breath tests to rule out carbohydrate malabsorption and small intestinal bacterial overgrowth
In cases of severe symptoms that may indicate gastroparesis	Gastric emptying scintigraphy and ^13^C breath tests (^13^C‐octanoic acid and ^13^C‐acetate)
In case of severe symptoms which suggest impaired gastric sensitivity and accommodation	Barostat and drinking test (water and liquid test meal)
In case of severe symptoms which suggest generalized gastrointestinal motility disorders	Extended motility testing (esophageal manometry, antroduodenal manometry, and small bowel manometry)
If there is evidence of systemic disease	Extended laboratory testing
In cases of very restrictive eating behavior	Consider an eating disorder. Questionnaires such as the five‐question SCOFF screening questionnaire may help to identify these.

## 5. General Measures

The treatment of FD is challenging due to its multifactorial causes. Given its fundamentally benign nature and the lack of effective therapies, the therapeutic approach is based on interdisciplinary collaboration and pharmacological treatments. Before initiating therapy, a careful diagnosis is required, which also includes comprehensive patient information to establish an empathic trustful relationship.

For patients with dyspeptic complaints, it is important to discuss the reasons for their consultation before diagnostic and therapeutic approaches. While some patients seek treatment because their quality of life is significantly reduced by their symptoms, others worry that the symptoms could indicate a life‐threatening condition.

There is widespread agreement that lifestyle and dietary changes are effective for FD, although there are no prospective studies on this. High‐fat meals cause more severe nausea and pain compared to meals high in carbohydrates. Quitting smoking is a helpful lifestyle modification [62, 63].

According to the current Rome IV criteria, FD is divided into two subgroups based on the main symptoms: patients with EPS, that is, predominantly upper abdominal pain or burning, and patients with PDS, who predominantly complain of a feeling of fullness and early satiation.

Treatment strategies for these patient groups do not differ significantly, and treatment goals should be determined and agreed upon in consultation with the concerned patient. It is important to communicate realistic expectations regarding treatment outcomes and to clarify that it is unlikely that any specific treatment will completely eliminate symptoms in the short term and in the long term.

Instead, the goal is to develop strategies together with the concerned patient to adequately control symptoms. Lifestyle changes that take into account factors such as dietary habits, stress, and shift work, as well as dietary approaches aiming at avoiding symptom‐causing foods (e.g., alcoholic beverages and gluten‐containing foods), should be considered.

Patients with FD have a significantly reduced physical fitness level compared to healthy individuals [64]. At the same time, initial data suggest that moderate exercise is effective in patients with FD [65]. Therefore, regular cardiovascular exercise can also be recommended as part of general approaches. This exercise should be adapted to individual abilities, but data are currently lacking to determine the optimal duration and intensity. All of these general approaches should be considered. Drug treatment depends mainly on the primary symptoms and their severity.

Statements–Recommendations

5.1. An empathetic approach and a detailed explanation of diagnostic and therapeutic approaches are important.

5.2. Counseling on a suitable lifestyle, which should be implemented independently, should be implemented prior to further therapeutic activities.

## 6. Psychoeducation and Psychotherapy for Patients With FD

According to the biopsychosocial model, biological, psychological, and social factors are involved in the pathogenesis of FD, which, in individual combination, can contribute to the development and maintenance of the disease [35]. Against this background, it is not surprising that psychological and social factors should also play a role in therapy [66]. An important aspect of treatment is successful communication between medical staff and patients, as well as a sustainable relationship between both groups [67]. This involves communicating the diagnosis (as a preliminary diagnosis) at an early stage and then identifying pathogenetically relevant factors and incorporating them into an overall biopsychosocial construct [66]. Last but not least, providing information about the disease, its progression, and the treatment options in the context of psychoeducation is of crucial importance, though relevance of this factor is difficult to show in clinical studies. A recent study in over 500 physicians, which included more than 5300 physician–patient consultations, found that patients with FGID more frequently (55.4%) attributed their symptoms to food or other somatic causes (43.6%), while physicians suspected psychosocial stressors as the cause in 65.4% of cases [67]. These were then addressed in a conversation in 70.8% of cases, and in about 10% of cases psychotherapy was finally recommended [67].

A 2021 systematic review including a meta‐analysis compiled the current data on the use of psychotherapy in patients with FD and included nine randomized controlled trials. Although these studies were relatively small, single‐center, and heterogeneous, due to the psychotherapeutic techniques used, they demonstrated across the studies an improvement in global symptoms (standardized mean difference −1.33, 95% confidence interval −1.97 to −0.68) of FD by employing psychotherapy [68]. A recent meta‐analysis also showed positive effects on the quality of life and lowering anxiety in patients with FD [69].

### 6.1. Select the Suitable Psychotherapeutic Procedure

Traditionally, and also for FD, most data exist on cognitive behavioral therapy, which uses techniques such as cognitive restructuring, coping strategies, and activation of resources. Cognitive behavioral therapy has been shown to result in improvements in symptom severity [70–75], pain intensity [73], and perceived illness‐related impact [76]. These positive effects were recently confirmed in a systematic review of seven studies [77].

Psychodynamic psychotherapy, which addresses intrapsychic conflicts and the resulting interpersonal difficulties, has also demonstrated positive effects on symptoms of FD [78–80].

Last but not least, hypnotherapy, which suggests relaxed and pleasant states, has also been shown to have positive effects in terms of symptom relief [81] and improved quality of life in patients with FD [82]. This intervention can also be offered in a resource‐saving manner using audio‐supported self‐treatment, as recently shown in a pilot study [83]. Positive effects on symptoms, quality of life, anxiety, and stress were observed [83]. These results should be replicated in a larger controlled study.

Hypnosis is an evidence‐based treatment option for IBS, whereas hypnosis for FD is not well established and has been less studied. Calvert et al. randomized 126 patients with FD for 16 weeks to hypnosis, supportive therapy plus placebo, or drug treatment with ranitidine 300 mg/day. Symptom changes were recorded both in the short term (16 weeks) and long term (56 weeks) [82]. Quality of life was also measured as a secondary endpoint. Hypnosis was induced by eye fixation, followed by progressive muscle relaxation, and deepened using standard procedures. At short‐term follow‐up, hypnotherapy was significantly more effective than both supportive and drug treatment in both symptom improvement and quality of life scores; this effect continued for long‐term follow‐up over 56 weeks. Furthermore, consultations with the general practitioner due to dyspepsia symptoms were significantly reduced. The authors concluded that hypnotherapy is a potent and cost‐effective treatment for FD.

In another study, Chiarioni et al. investigated gastric emptying using ultrasound and epigastric symptoms in 11 healthy subjects and 15 patients with FD under three conditions: (a) basal, (b) after cisapride 10 mg, and (c) during 90 min of gut‐directed hypnosis therapy. Hypnosis was significantly more effective than cisapride and relaxing music in reducing gastric emptying time in both patients with FD and healthy volunteers (p < 0.005) [84]. Dyspeptic symptoms were also significantly improved by hypnosis in patients with FD, although no correlation with gastric emptying time was found. A single session of hypnosis was as effective as prokinetic treatment in FD, but the mechanism due to which symptoms improvement remains unclear [85].

Statements–Recommendations

6.1. The pathogenesis of FD is multifactorial and can best be explained by the biopsychosocial model.

6.2. Psychoeducation plays a role at the beginning of FD therapy but has not been investigated as an isolated intervention.

6.3. Before treatment, the treatment goals should be detailed. Reducing symptoms and improving quality of life are important goals.

6.4. There are favorable data for psychotherapy in FD, as shown in several meta‐analyses, but the heterogeneity of the studies must be taken into account.

6.5. Hypnotherapeutic procedures can be used, even in self‐treatment, but research on this is heterogeneous.

## 7. Drug Therapy

Because the pathophysiology of FD is multifactorial and the underlying symptom‐causing disorders are heterogeneous, many drug treatment trials demonstrate efficacy only in subgroups of affected individuals. Thus, the overall results in clinical trials are often inconclusive, and the body of evidence for most therapies is weak. In general, the effectiveness of all treatment options varies largely from individual to individual and is difficult to predict. Therefore, every treatment is always probatory in nature and should be discontinued after 3 months at the latest if it is ineffective.

### 7.1. Drug Therapy Options

#### 7.1.1. *Helicobacter* Eradication in HP‐Positive Patients

A number of studies have shown that *HP* eradication in *HP*‐positive patients can have, on average, a moderate effect on symptoms (effective in approximately 10%–15%) [86] and in some patients can even lead to a complete cure of FD symptoms [87]. This healing can take up to 1 year after *HP* eradication.

#### 7.1.2. Phytotherapy

The phytotherapeutics STW‐5/STW‐5‐II and peppermint oil in combination with caraway oil can alleviate the symptoms of FD. The available studies from German‐speaking countries are considered broad. Given their efficacy and the limited availability of other treatment options, phytotherapeutics are considered first‐line treatments for the indication FD. There is only limited evidence for other herbal therapies; these may be used as a secondary option if their mechanisms of action are appropriate for the targeted symptoms.

#### 7.1.3. STW‐5/STW‐5‐II

Meta‐analyses [88–94], analyzing data from various small RCTs, showed that the combination of *Iberis amara*, peppermint, chamomile, caraway, lemon balm, licorice, angelica root, milk thistle, and greater celandine (=STW‐5) was superior to placebo in the treatment of FD symptoms. No serious side effects were observed. A recent meta‐analysis [95–97] showed for STW‐5‐II (*Iberis amara*, chamomile, caraway, lemon balm, peppermint, and licorice) an overall improvement in the symptoms of patients with FD and additionally significant improvements in the feeling of fullness, premature satiation, and upper abdominal pain compared to placebo after 4 and 8 weeks. Symptoms such as abdominal cramps, belching, loss of appetite, and retrosternal discomfort were also significantly alleviated.

#### 7.1.4. Peppermint Oil/Caraway Oil

Two reviews/meta‐analyses [98, 99] of five clinical trials on peppermint oil in combination with caraway oil (five RCTs [100–105]) show that the combination significantly improves the global symptoms of FD (pain intensity and Clinical Global Impression Scale) in the medium and long term and does so with a similar safety compared to a placebo.

#### 7.1.5. Acid Suppression

• PPIs, for example, omeprazole, pantoprazole, esomeprazole, lansoprazole, and rabeprazole [106]
• H2 receptor antagonists: for example, famotidine, cimetidine, and ranitidine [107]
• If necessary, alginates, antacids (no direct study evidence for these substances, but since the concept of acid suppression works in many patients, they can be considered an alternative or complementary treatment option given their good tolerability).

The effects of PPIs have been investigated in a number of controlled trials and summarized in meta‐analyses [106]. In 25 randomized trials involving more than 8000 patients with FD, PPIs were minimally more effective than placebo in relieving global dyspepsia symptoms when acid‐associated symptoms such as heartburn or regurgitation were among the major symptoms. According to the meta‐analysis and the individual studies, PPI therapy is not effective in FD without acid‐related symptoms. The difference to H2 receptor antagonists was not significant.

A Cochrane review on the treatment of FD with H2 receptor antagonists did not find any efficacy in FD patients according to Rome III criteria [108].

Small studies that do not meet today′s quality standards show that antacids (sucralfate) are more effective compared to placebo treatment [109]. Alginates in combination with ranitidine have been shown to alleviate EPS‐type FD symptoms; the study found overall better symptom control for PPI treatment (40% vs. 61%) [110].

In patients with FD who respond to PPI treatment, the suspected underlying reflux pathophysiology of the symptoms should be explained, and an approach should be made to slowly taper off the PPIs after a few weeks, in order to avoid adverse effects of PPI therapy or acid rebound [111]. In Germany, PPIs are not approved for the indication FD, so their use would be off‐label.

#### 7.1.6. Gastroprokinetics: Metoclopramide and Domperidone

Two meta‐analyses demonstrate the effectiveness of prokinetics in FD [112, 113]. Some of the substances examined are not approved in Germany (see below). The data for the old substances metoclopramide and domperidone are of low quality, but in daily clinical practice their use has proven being successful, at least for short‐term use [114–116]. In Germany, these prokinetics are not approved for the indication FD and are no longer approved for long‐term treatment in other indications. QT prolongation with the risk of arrhythmias up to torsade de pointes tachycardia and neurological side effects are possible; tachyphylaxis is known.

#### 7.1.7. Simethicone

The carminative simethicone reduces the surface tension of gas bubbles. Simethicone is suitable for improving the symptoms of FD; a placebo‐controlled study including 185 patients with FD showed that simethicone (three times daily) was superior to placebo at all evaluation time points, after 2, 4, and 8 weeks, in terms of relief of FD symptoms. A third study arm investigated the efficacy of the prokinetic cisapride. In Week 2, simethicone was also superior to cisapride in efficacy, but in Weeks 4 and 8, simethicone was not found to be significantly different in efficacy compared to cisapride [117].

### 7.2. Second‐Line Treatments

Neuromodulators are used in the treatment of FD due to their effectiveness in modulating pain and reducing neuronal communication in the gut–brain axis. Among other things, due to the increased potential for side effects (including sedation and dry mouth), these substances are more likely to be used as second‐line treatment for persistent, treatment‐resistant symptoms. Explaining the mechanism of action of FD to the patient can increase therapeutic success.

#### 7.2.1. Tricyclic Antidepressants

Tricyclic antidepressants (TCAs, low doses) have long been used as co‐analgesics for the treatment of chronic pain. The effectiveness of TCAs such as amitriptyline, nortryptiline, and desipramine has also been demonstrated in functional gastrointestinal symptoms [118, 119]. For use in FD, low doses of 10–50 mg are chosen. These are taken in the evening and increased in small increments every 7–14 days, depending on response, up to the maximum dose. Typical side effects include constipation, fatigue, and dry mouth. In addition to amitriptyline, the tricyclic antidepressants nortriptyline and desipramine are also used in the treatment of FD. Effectiveness or ineffectiveness can be assessed after 8–12 weeks. No studies are available on the long‐term use in FD therapy. Thus, a tapering regimen can be recommended after achieving stable remission for 6 months.

#### 7.2.2. Mirtazapine

This antidepressant from the group of tetracyclic antidepressants is known for its appetite stimulation and can therefore be particularly helpful in cases of accompanying loss of appetite. Efficacy has been demonstrated in patients with FD and weight loss [120]. Dosages of 7.5–45 mg are used for FD, starting at a low dose and increasing based on response. It is taken in the evening. Explaining the mechanism of action of FD to the patient can increase therapeutic success. Typical side effects include increased appetite with weight gain, fatigue, headache, and dry mouth.

#### 7.2.3. Sulpiride

Sulpiride is an atypical neuroleptic that also has a gastroprokinetic effect and can relieve dyspepsia symptoms [119, 121].

#### 7.2.4. Other Second‐Line Therapeutics

For other substances from the group of prokinetics, efficacy in FD has been proven in studies, but these are currently not approved in Germany [119]. These include acotiamide (fundus relaxant and prokinetic effect), itopride (prokinetic effect), mosapride (prokinetic effect), and cinetapride (prokinetic effect).

#### 7.2.5. Drug Therapies Not Recommended

Analgesics such as nonsteroidal anti‐inflammatory drugs, paracetamol, cannabinoids, or opioids should not be routinely used to treat FD. When using cannabinoids for recreational purposes, it should be noted that cannabinoids can cause clinical symptoms similar to dyspepsia with nausea, and in more severe cases even hyperemesis or cyclic vomiting [122–124].

Statements–Recommendations

7.1. Symptom improvement with medication often only occurs after 8–12 weeks of treatment. It is important to remind patients that patience is required. Likewise, medications that are ineffective should be discontinued.

7.2. In drug therapy, there are positive clinical studies on prokinetics, *HP* eradication, phytotherapeutics, and, if heartburn and regurgitation are the major symptoms, on acid suppression with PPIs and H2 receptor antagonists. Efficacy of PPI and H2 receptor antagonists occurs for acid‐related symptoms such as heartburn and regurgitation.

7.3. The phytotherapeutics STW‐5/STW‐5‐II and peppermint oil in combination with caraway oil are considered being first‐line therapies and can alleviate the symptoms of FD and should be considered for patients with FD.

7.4. The phytotherapeutic formula Rikkunshito from Kampo medicine and special formulas from Traditional Chinese Herbal Medicine can alleviate the symptoms of FD. Chinese herbs of assured quality are currently not sufficiently available in Germany.

7.5. PPIs, H2 receptor antagonists, and prokinetics are not approved for the indication FD in Germany; their use would be off‐label.

7.6. *HP* eradication therapy should be considered in FD with HP infection.

7.7. In cases of *HP* negativity, PPIs are an option if acid‐associated symptoms are predominant.

7.8. Prokinetics such as domperidone or metoclopramide may be used, provided that there are no contraindications. Prokinetics such as metoclopramide and domperidone should not be used for long‐term treatment. In Germany, these active ingredients are not approved for FD.

7.9. In second‐line drug therapy, there are positive clinical studies on various neuromodulators such as amitriptyline, mirtazapine, and sulpiride.

7.10. Amitriptyline may be considered for patients with persistent symptoms. Treatment for FD begins with a low dose (e.g., 10 mg at bedtime). The dosage is then slowly increased to a maximum of 50 mg depending on response and tolerability. A treatment trial of at least 12 weeks should be discussed.

## 8. Non‐Drug Therapy Options

### 8.1. Mind–Body Techniques

There are initial promising studies on the positive effects of stress management training, mindfulness‐based therapy, and diaphragmatic breathing training on FD, but their effectiveness and long‐term effects cannot be conclusively assessed as of now [46, 125–129].

### 8.2. Traditional Medicines

#### 8.2.1. Japanese Herbal Medicine (KAMPO)

The phytotherapeutic formulation Rikkunshito from Kampo medicine can alleviate the symptoms of FD. Rikkunshito is a phytotherapeutic agent commonly used for FD in Asia. There is high‐quality evidence that Rikkunshito can improve functional disorders of the gastrointestinal tract [130]. The Japanese guideline [131] on FD contains a strong recommendation for Rikkunshito, but this is not sufficiently supported by the available data.

#### 8.2.2. Traditional Chinese Medicine (TCM)

Manual and electroacupuncture: Acupuncture has traditionally been used in Eastern countries to treat FD, although the underlying mechanisms remain unclear [132, 133]. Ma et al. conducted a randomized controlled trial to investigate the efficacy of acupuncture in FD compared with the prokinetic itopride [134]. In the study, 712 patients with FD were randomly assigned to either acupuncture, sham acupuncture, or the drug control group (itopride) for 4 weeks. The overall response rate was significantly higher in the acupuncture group (70.7%) compared to the other groups, with the smallest effect observed in the sham acupuncture group (34.8%). Zeng et al. recently investigated brain responses to acupuncture in FD [135]. They demonstrated that the acupuncture group showed a comprehensive deactivation of brain activity compared to the sham acupuncture group. This deactivation also correlated with symptom improvement, suggesting the potential efficacy of acupuncture at specific points in FD. These effects are further supported by a recent PET‐CT study [136].

Several meta‐analyses, which identified numerous low‐quality RCTs, showed that manual and electroacupuncture can be effective in the treatment of FD [137–139]. This was demonstrated by improvements in symptoms and health‐related quality of life. A recent meta‐analysis [140] shows that various types of acupuncture combined with western medicine are significantly more effective in improving symptoms of FD than western medicine alone. Superior efficacy was demonstrated in relieving early satiation and postprandial fullness symptoms. For the relief of epigastric pain, acupuncture combined with moxibustion proved to be the most effective treatment, while moxibustion emerged as the optimal choice for treating retrosternal burning. The interpretation of the results within the meta‐analyses is complicated by the generally low number of included patients, different acupuncture application protocols, selection bias, performance bias, attrition bias, reporting bias, and blinding.

Few studies conducted in western countries have not produced positive outcome data, so a Cochrane review also concludes that it is not certain whether manual acupuncture or electroacupuncture is more effective or safer than other treatments for patients with FD [138, 141].

#### 8.2.3. Traditional Chinese Herbal Medicine

An umbrella review [142] of systematic reviews regarding the efficacy of TCM in FD identified numerous low‐quality, low‐patient‐number RCTs showing that various TCM preparations, alone or in combination with various prokinetic medications, may be effective and superior to prokinetic medication alone. Three specific formulations appeared to show better results in relieving global dyspeptic symptoms: Si Ni San, modified Xiao Yao San, and Xiang Sha Liu Jun Zi Decoction. TCM could be considered a treatment alternative when prokinetic agents and PPIs are contraindicated. No serious adverse events were reported in the studies [143] conducted. Chinese herbs of sufficient quality are currently unavailable in Germany.

Statements–Recommendations

8.1. There are initial promising studies on the positive effects of stress management training, mindfulness‐based therapy, and diaphragmatic breathing training on FD, but their effectiveness and long‐term effects cannot yet be conclusively assessed.

8.2. Manual and electroacupuncture can be effective in treating FD.

## 9. Dietary Modification

Although 80% of patients show typical symptoms of FD after specific food triggers and meet the Rome IV criteria for PDS [6, 144–146], a systematic review of 16 studies could not derive any evidence of an association between symptoms of FD and food intake [147, 148]. Also, there are no randomized controlled trials of dietary interventions in patients with FD.

Even in healthy individuals, food intake may cause characteristic gastrointestinal effects. Food intake releases gastric acid and hormones, alters gastric accommodation and gastroduodenal motility, activates the mucosal immune system, and can influence peripheral and central pain perception and the microbiome [147, 149, 150]. These effects appear to play a role in the genesis of FD‐associated symptoms.

Food‐related symptoms usually begin 15–30 min after eating and can last for several hours. They characteristically occur sequentially, first with a feeling of fullness and bloating, followed by nausea, and later with epigastric pain and burning [144]. The most common symptom triggers are fatty foods, alcohol, coffee, red meat, carbonated drinks, hot and spicy foods, citrus fruits, dairy products, vegetables, and wheat [35]. For some of the trigger factors mentioned, there is discrete evidence suggesting a causal relationship. For example, duodenal lipid infusions physiologically stimulate CCK release, leading to delayed gastric emptying. In patients with FD, this can increase visceral hypersensitivity [151]. Long‐chain triglycerides play a particularly important role in the exacerbation of functional symptoms, inducing symptoms such as bloating and nausea more strongly than medium‐chain triglycerides or glucose [152]. Irregular and rapid eating is associated with an increase in dyspeptic symptoms [153].

The research on alcohol is inconsistent. Beer and wine, as well as regular and increasing alcohol consumption, are more likely to be associated with symptoms, suggesting that abstinence or reduction in alcohol consumption could have a positive effect on symptoms [154–157]. Caffeine triggers symptoms in up to 50% of patients [147]. Many patients with FD exhibit hypersensitivity to capsaicin, a main component of spicy foods, which decreases with regular consumption [158, 159]. Milk, grains, and vegetables are a major component of fermentable oligo‐, di‐, monosaccharides, and polyols (FODMAP).

Lactose intolerance should be ruled out if medical history points towards it. Since both lactose intolerance and fructose malabsorption are common, they should be tested. There are no studies or general recommendations that a lactose‐free or fructose‐reduced diet can improve FD symptoms without confirmed intolerance.

Eating five servings of seasonal fruits and vegetables is recommended for a balanced diet. Patients with IBS benefit from eating cooked vegetables instead of raw vegetables. It has not been proven yet, if this is beneficial in FD as well. Every second person with FD reports symptoms after consuming wheat [160]. However, the prevalence of celiac disease is not higher as compared to prevalence in healthy individuals [161]. While a gluten‐free diet (open‐label study with n = 22) improved symptoms in 80% of patients, only 25% of patients subsequently responded with symptoms to a double‐blind gluten challenge [162].

The overall concept of a low‐FODMAP diet was also evaluated in FD. A small open‐label study showed that eliminating high‐FODMAP foods for 4 weeks was more effective in alleviating symptoms compared to traditional dietary recommendations (reduction of caffeine, alcohol, fat, fiber, and dietary supplements) (response rate 50% vs. 16%) [163]. Patients with IBS or an overlap of both conditions were also included. However, there are no long‐term data available. Follow‐up studies have not been able to reproduce any significant effects of a low‐FODMAP diet on FD symptoms, either in the short or the long term [164]. It is possible that patients with EPS may respond better. Overall, there is sufficient evidence that a low‐FODMAP diet cannot be recommended for the treatment of FD.

Many affected individuals attempted to change their diet by eating more frequent, smaller, and lower‐fat meals compared to healthy individuals [165]. Very restrictive diets can lead to malnutrition and disordered eating behavior. However, there is evidence that up to 50% of those diagnosed with FD may also have an underlying avoidant/restrictive food intake disorder (ARFID) [166]. In cases of severe weight loss, severe symptoms, or a treatment‐resistant course of the disease, nutritional counseling is recommended.

The multifactorial etiology of FD requires multimodal therapy. Individualized nutritional therapy can therefore be attempted as part of a multimodal therapeutic approach [149].

Statements–Recommendations

9.1. Most patients associate their symptoms with food intake. Studies do not support this. Common trigger factors cited by patients include high‐fat foods, alcohol, coffee, carbonated beverages, capsaicin, vegetables, citrus fruits, carbohydrates, and wheat.

9.2. Frequent, smaller meals and avoiding high‐fat foods appear to help. The low‐FODMAP diet has no role in FD.

9.3. Individualized nutritional counseling should be integrated into multimodal therapy.

## 10. Microbiome Therapy

A wide variety of factors, such as lifestyle, dietary factors, individual hygiene, and medication intake, influence the gastrointestinal microbiota. Apart from postinfectious FD and *HP*‐associated dyspeptic symptoms, little is known about the pathophysiology of FD and the gastrointestinal microbiota.

### 10.1. Antibiotics

Antimicrobial therapy against *HP* is recommended for patients with FD and has been discussed previously.

Gut‐selective antimicrobial therapy with rifaximin is effective in FD, even in the absence of *HP* infection [167]. A placebo‐controlled study in which 86 patients were enrolled and randomized according to Rome III criteria showed that 400 mg rifaximin *compared to* placebo for 14 days improved the global symptom score after 8 weeks, but not after 4 weeks. The secondary endpoints of belching and postprandial fullness were significantly improved compared to placebo after 4 weeks. This supports the notion that microbial factors cause symptoms in at least some patients with FD. Given the latency of rifaximin intake and efficacy, further studies are required [168–170].

### 10.2. Probiotics

Based on the assumption that microbiome‐associated factors may play a role in the pathophysiology and treatment of FD, therapeutic studies have been conducted with probiotics, probiotic foods, and various types of postbiotics in FD [171, 172]. Given that little is known about the potential role of the microbiota in FD, the assessment of the usefulness of such therapeutic approaches is limited. Therapeutic studies on probiotics, probiotic foods, and postbiotics in patients with FD have a small number of subjects and are preliminary in nature, but they demonstrate that therapeutic effects can potentially be achieved with individual microbial preparations [171–173]. Analogous to the evaluation of probiotic therapies in the treatment of symptoms of IBS, a probationary, time‐limited therapeutic trial with re‐evaluation after 4 or 6 weeks seems conceivable. Identifying effective microbes or promising therapeutic success does not seem appropriate given the current study situation.

Statements–Recommendations

10.1. There is insufficient information available to evaluate the treatment of FD with antibiotics.

10.2. There is not yet sufficient information available to evaluate the treatment of FD with probiotics.

## 11. Other Therapy Options

In the absence of a causal therapeutic approach for FD, patients and physicians often choose therapies for which there is no evidence or only limited evidence in the literature. Recommendations, especially from a physician′s perspective, are largely based on positive experience in routine clinical practice. In the following paragraphs, therapies for which the evidence is insufficient to make a positive guideline recommendation are described, even if individual data are available in the literature.

Probatory use of enzymes is not recommended in the guidelines for IBS. Older clinical data are available for the treatment of patients with FD with digestive enzymes. The clinical effect of a fixed combination of gastric mucosal extract and amino acid hydrochlorides used is not aimed to substitution, but rather to support the proteolytic release of amino acids. A randomized, placebo‐controlled, double‐blind, crossover study in 1167 patients (1) demonstrated significant efficacy in reducing individual dyspeptic symptoms (p < 0.001) [174].

Capsaicin is traditionally used to treat various pain syndromes due to its ability to selectively impair the action of pain‐sensitive fibers.

Red pepper powder (*Capsicum annuum*) was more effective than placebo in improving dyspeptic symptoms in a small randomized controlled trial (n = 30) [175–177]. The authors speculated about a potential effect on visceral gastric pain perception, but no further studies have yet replicated these data. Ginger (*Zingiber officinale*) is also traditionally used to treat dyspeptic symptoms.

Recently, the effect of ginger on gastric sensorimotor function was investigated in 11 patients with FD. In this small, open‐label study, ginger demonstrated prokinetic effects but had no effect on gastric sensation, dyspeptic symptoms, or intestinal peptides/hormones [178, 179].

Artichoke (*Cynara scolymus*) leaf extracts are commonly used to treat dyspeptic symptoms. The bitter compounds (cynaropicrin) are believed to increase bile flow and have hepatoprotective, antioxidant, and antispasmodic effects [180–182]. In a multicenter, double‐blind, randomized controlled trial, 247 patients with FD were treated with either a commercial artichoke leaf extract (ALE) or placebo [20]. After 6 weeks, the ALE preparation was significantly more effective than placebo in the intention‐to‐treat analysis in relieving symptoms (p < 0.001) and improving the quality of life index in patients with FD [183].

Less well‐studied therapeutic approaches such as a combination of simethicone, magnesium oxide, and charcoal have been shown to improve bloating, epigastric burning, and pain in a placebo‐controlled trial of 276 patients [184].

Statements–Recommendations

11.1 If treatment options with strong evidence are not sufficiently effective, options with low evidence can be used. Examples include enzyme therapy and other herbal remedies such as capsaicin, ginger, or artichoke extract.

## 12. Treatment Algorithm

**Figure 2:**
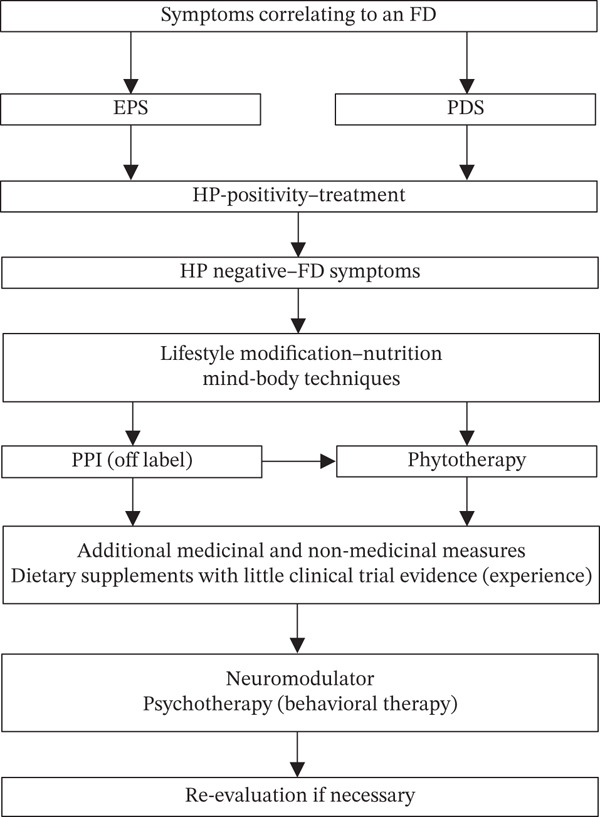
Treatment algorithm for functional dyspepsia.

The treatment algorithm for FD is shown in Figure 2.

## 13. Course and Prognosis

The course of FD can be chronic or recurrent [185]. Many patients experience recurring symptoms over months or years, which can greatly vary in frequency and intensity. This can be individually related to stress, dietary habits, or other factors. A typical feature of FD is that it can fluctuate in intensity, so that patients experience symptom‐free phases followed by symptomatic flare‐ups.

In practice, two common courses of illness are observed in many patients: First, there are patients in whom symptoms remain largely constant over years, often leading to a significant impairment of quality of life. Second, there are also patients in whom symptoms become milder over time or manifest themselves in phases of symptom‐freeness.

Psychological stress, in particular, can have a significant impact on the course of the disease. Patients often report worsening symptoms during periods of emotional stress or psychological strain, indicating that psychosocial factors can play a significant role in the development and progression of dyspepsia.

The prognosis of FD varies considerably depending on the individual case, and common patterns or symptom clusters have not been described, apart from the overarching classification into EPS and PDS. Many patients experience recurring symptoms over the years, sometimes more severe, sometimes less severe. A complete cure is usually not possible, but targeted treatment can alleviate symptoms and improve quality of life for many patients. Therefore, symptom relief and improved quality of life should be considered central aspects of the therapeutic approach.

In population‐based studies over 10–12 years, approximately 20% of patients had persistent symptoms, and 40%–50% of patients experienced resolution of symptoms. Accordingly, the long‐term prognosis for patients with FD is considered positive; 30%–35% of patients had fluctuating symptoms, or a transition to another functional gastrointestinal disorder was evident [186].

The life expectancy of patients with FD is not shortened [187]. The long‐term prognosis depends on various individual factors, such as the severity of symptoms, the individual′s approach to the disease, and treatment adjustments. Early detection of the disease, diagnosis, and communication of the diagnosis, as well as a holistic approach to treatment that considers both physical and psychosocial aspects, can positively influence the course of the disease.

Statements–Recommendations

13.1. The course of FD can be variable, with persistent or episodically intermittent symptoms.

13.2. In a large proportion of patients with FD, the long‐term outcome is positive.

13.3. The life expectancy of patients with FD is not limited.

### 13.1. Methods

In the German AWMF guideline system, the classifications S1, S2, and S3 reflect different levels of methodological rigor. S1 guidelines are based on an informal expert consensus without a structured consensus method or a systematic literature search. S2 guidelines exist in two forms: S2k, which relies on a structured and moderated consensus process but does not require a systematic literature review, and S2e, which is grounded in systematic evidence search and appraisal but does not include a formal consensus procedure. S3 guidelines represent the highest methodological standard, as they combine both a systematic review of the evidence and a formalized, structured consensus process involving multidisciplinary stakeholders. The manuscript presented here is an S1 guideline, does reflect daily clinical practice, and its recommendations are therefore derived from expert consensus without additional levels of evidence given.

**Nomenclature**
ALE: artichoke leaf extract
ARVID: avoidant/restrictive food intake disorder
CCK: cholecystokinin
DGBI: disorder of gut–brain interaction
DGNM: German Society for Neurogastroenterology and Motility
EPS: epigastric pain syndrome
FD: functional dyspepsia
FGID: functional gastrointestinal disorders
FODMAP: fermentable oligo‐, di‐, monosaccharides, and polyols
HP: *Helicobacter pylori*
NNT: number needed to treat
EGD: esophagogastroduodenoscopy
PDS: postprandial distress syndrome
PPI: proton pump inhibitor
PPV: positive predictive value
RCT: randomized clinical trial
TCA: tricyclic antidepressants
TCM: Traditional Chinese Medicine

## Funding

No funding was received for this manuscript. Open Access funding enabled and organized by Projekt DEAL.

## Disclosure

This review/recommendation was previuosly published in a German language version in Zeitschrift für Gastroenterologie, 2025; 63: 403–422 | 2025. [188]

## Conflicts of Interest

S.S. Honorariums for lectures, Advisory Board: Der Campus, Fortbildungskolleg, Medfora, MEDICE Arzneimittel Pütter GmbH & Co. KG, Weber & Weber, Repha GmbH, Synformulas, Dr. Wilmar Schwabe GmbH & Co. KG, Falk Foundation e. V., Reckitt Benckiser Deutschland GmbH, Bayer Vital GmbH; T.F. Honorariums for lectures, Advisory Board: Dr. Wilmar Schwabe GmbH & Co. KG, Sanofi‐Aventis Deutschland GmbH, Takeda Pharma Vertriebs GmbH & Co. KG, Falk Foundation e. V., Medical Forum, Forum for Medical Continuing Education, Promedia Medical Technology, MEDICE Arzneimittel Pütter GmbH & Co. KG, ABOCA Group SpA, Reckitt Benckiser Deutschland GmbH, Bayer Vital GmbH, nutrimmun GmbH; J.G. Honorariums for lectures, Advisory Board: Campus, CGC, Daiichi Sankyo, Esanum, Fortbildungskolleg, Galapagos, Isgro, Luvos, Medical Tribune, Medfora, MediBayern, Meusel Healthcare, Microbiotica, Norgine, Nutrimmun, Pileje, Repha, RG, Signum, Synformulas, Takeda, Themessenger; J.K. Honorariums for lectures, Advisory Board: Falk, GE Healthcare, Takeda, Enterra, Medtronic, Mylan, Repha GmbH, Standard Instruments, Takeda; J.L. Research Support: Steigerwald Arzneimittelwerke GmbH, Falk Foundation, TechLab, Inc., Dr. Willmar Schwabe, Repha GmbH biological medicinal products, Honorariums for lectures, Advisory Board: AbbVie Deutschland GmbH, ABF‐Synergie GmbH, AlphaSigma, Bionorica SE, Bristol‐Meyer Squibb, Enterosan Labordiagnostik, Falk Foundation; Galapagos Pharma, Janssen Cilag, Loges + Co. GmbH, Luvos Just GmbH, Mauna Kea, Dr. Pfleger Arzneimittel, Pfitzer Phrama GmbH, Repha GmbH; C.P. Honorariums for lectures, Advisory Board: Falk Foundation e. V.; A.M. Honorariums for lectures, Advisory Board: Dr. Wilmar Schwabe GmbH & Co. KG, Falk Foundation e. V., Reckitt Benckiser Deutschland GmbH, Bayer Vital GmbH; A.S. Conflict of interest: The authors state that there are no conflicts of interest.

## Data Availability

Data sharing is not applicable to this article as no datasets were generated or analyzed during the current study.
